# Positive selection of IL‐33 in adaptive immunity of domestic Chinese goats

**DOI:** 10.1002/ece3.2813

**Published:** 2017-02-23

**Authors:** Akhtar Rasool Asif, Muhammad Awais, Sumayyah Qadri, Hafiz Ishfaq Ahmad, Xiaoyong Du

**Affiliations:** ^1^ Key Lab of Animal GeneticsBreeding and Reproduction of Ministry EducationCollege of Animal Science and TechnologyHuazhong Agricultural UniversityWuhanChina; ^2^ College of InformaticsHuazhong Agricultural UniversityWuhanChina; ^3^ College of Veterinary & Animal Sciences (CVAS)JhangPakistan; ^4^ University of Veterinary & Animal SciencesLahorePakistan; ^5^ State Key Laboratory of Agricultural MicrobiologyCollege of Veterinary MedicineHuazhong Agricultural UniversityWuhanChina; ^6^ The Cooperative Innovation Center for Sustainable Pig ProductionWuhanChina

**Keywords:** evolution, goat, IL‐33, positive selection

## Abstract

The identification of the candidate genes that play key role in phenotypic variation in livestock populations can provide new information about evolution and positive selection. IL‐33 (71954) (Interleukin) gene is associated with the increased nematode resistance in small ruminants; however, the role of IL‐33 for the genetic control of different diseases in Chinese goat breeds is poorly described in scientific literature. Therefore, the current investigation was performed for the better understanding of the molecular evolution and the positive selection of single‐nucleotide polymorphism in IL‐33 gene. Fixation Index (*F*_ST_)‐based method was used for the outlier loci determination and found that IL‐33 was present in outlier area with the provisional combined allocation of mean heterozygosity and *F*_ST_. Positively selected IL‐33 gene was significantly, that is, *p*(Simul *F*_ST_ < sample *F*_ST_ = 0.98*) present in corresponding positive selection area. Hence, our study provided novel information about the nucleotide variations in IL‐33 gene and found to be nonsynonymous which may helpful for the genetic control of diseases by enhancing the immune system in local Chinese goat breeds as well as in other analyzed vertebrate species.

## 1 Introduction

Recognition of signatures assortment is a significant tool to recognize possible genes that may trigger efficiently vital character and will recover our capability to connect inherited deviations to the significance of phenotype (Biegelmeyer, Gulias‐Gomes, Caetano, Steibel, & Cardoso, 2016; Consortium, 2009). From the previous centuries, cattle has been preferred intensively, such as it has attained remarkable phenotypic alteration from last 40 years. The selective breeding track is economically important based on the different traits of various genomic regions. Yet, it is unidentified how selections have altered the genome of Holstein and what types of changes in genome are linked with the phenotypic changes. The excess of polymorphism in genome sequence data (Nez et al., 2016) has provided a valuable inventive tool in the search for traces of the most recent selection in the genome, for example, (Biegelmeyer et al., 2016).

The human geneticists developed some statistical test to examine various assume deviation and unpredictable genetic under neutral model (Fumagalli et al., 2015). While all information is supported on impartial genomic deviation, not every of them rely on the equal class of sequence. The majority of these investigators were considered for full‐sequence data and not for genome extensive assortment of pre‐ascertained SNPs that are presently accessible in several livestock groups. A number of the primarily simply illustrious traces missing by the services of selection are those left by perceptive sweeps. Selective sweeps take place when an allele suit extra recurrent in a population as an effect of positive selection. As the positively selected allele enlarge in frequency, associated nearby alleles will act so, too, an occurrence known as genetic hitchhiking (Corbett‐Detig, Hartl, & Sackton, 2015).

IL‐33 in 2005 was recognized as a part of the IL‐1 family of cytokines (Schwartz et al., 2016). Currently, the family comprises of 11 members (Garlanda, Dinarello, & Mantovani, 2013). There is a good motivation for arrangement of these cytokines into a family. Commonly, most IL‐1 family elements are proinflammatory cytokines among pleiotropic roles in innate resistance. In addition, they contribute in determining adaptive immunity by skewing the isolation of naive helper T lymphocytes and by directly affecting the effecter functions of different subsets of T and B lymphocytes. IL‐33 acts on many different target cells in different organs. The role of IL‐33 has been recognized in numerous diseases, such as allergies, chronic inflammation of the gut (Lopetuso, Chowdhry, & Pizarro, 2013), especially allergic asthma (Liew, 2012; Saluja et al., 2015), cardiovascular diseases (Miller & Liew, 2011), disorders of the central nervous system (Gadani, Walsh, Smirnov, Zheng, & Kipnis, 2015), and rheumatoid arthritis (Palmer & Gabay, 2011). Similar to other IL‐1 family members, IL‐33 can be valuable or destructive, depending on the situation of the disease (Liew, Pitman, & McInnes, 2010; Palomo, Dietrich, Martin, Palmer, & Gabay, 2015).

The published scientific literature related to evolutionary positive selection studies on IL‐33 gene in goats is scarce. Positive selection in association with single‐nucleotide polymorphism (SNP) plus molecular evolution in IL‐33 gene is a significant phenomenon. Therefore, it is imperative to find out the nucleotide and genetic discrepancies. Henceforth, the current study presents unique data on nucleotide sequence change in IL‐33 gene in native breeds of Chinese goats along with other observed mammalian species.

## 2 Materials and Methods

### 2.1 Ethics statement

The entire experimental protocols were certified by the Law of Animal Husbandry in People's Republic of China (Dec 29, 2005). All the procedures and steps for ear tissue collection were assessed and approved by the Biological Studies Animal Care and Use Committee of National Animal Husbandry Service, Hubei, PR China. All the other factors were considered to alleviate any suffering and pain while taking these tissue samples.

### 2.2 Experimental animal selection and genomic DNA extraction

The study was conducted on 105 goats of four different breeds, namely Hybrid white yellow, Enshi black, Yichang white, and Nanjing yellow and had been selected from southern districts of China. Genomic DNA extraction kit TIANamp (TianGen, Beijing, China) was used for the extraction of genomic DNA from the ear tissue samples of experimental goats.

### 2.3 Genotyping/SNP sequencing

The genomic DNA for the extension of 10 anticipated SNPs locus was used for screening and characterization of these SNPs. SeqMan program was used for the sequences of these identified SNPs and then aligned and genotyped in 105 goats using Matrix‐assisted laser desorption/ionization time of flight mass spectrometry (MALDI‐TOF) assay [SquenomMassARRAY:emoji:, BioyongTechnologeies Inc. HK].

### 2.4 FDIST analysis

Regarding analysis and evaluation of selection effects, Lositan software (Beaumont & Nichols, 1996) was used. Through allelic frequency, *F*_ST_ and *p* values were estimated for each locus based on heterozygosity. Replications were designed comprised of four populations: 105 individuals, 21 SNPs, 10 loci, and an expected 0.102 *F*_ST_ value. The outlier with *F*_ST_ is more than estimated indicating efficient divergent selection through this method and reducing heterozygosity (Akey et al., 2004). Approximately, 100,000 replications were employed on real data that construct the datasets of population. Keeping confidence limits at 95%, quantiles were supposed for provisional *F*_ST_ joint distribution against mean heterozygosity. Outlier was defined based on those loci expressed outside the simulated neutral distribution with a characteristic differentiation behavior.

### 2.5 Sequence analysis

Coding sequences of twelve mammalian species were downloaded from GenBank and then aligned the protein sequences using MEGA6.0 program (Tamura, Stecher, Peterson, Filipski, & Kumar, 2013) by considering parameters set for default alignment thereafter applying manual adjustment. Positive selection and amino acid sites under selection were determined by maximum likelihood technique. Twenty four different models were used in computerized program of MEGA6.0 program. The BIC (Bayesian information criterion) model scores are measured to explain the pattern of substitution. For every model, maximum likelihood value (lnL), AICc value (Akaike information criterion, corrected), and the number of parameters (including branch lengths) are also calculated (Nei & Kumar, 2000). Gamma distribution (+G) with 5 rate categories and by assuming that a certain part of sites are evolutionarily invariable (+I). Estimated values of nucleotide frequencies (f), rates of base substitutions (r), and transition/transversion bias (R) for all nucleotide pair were calculated. In the final dataset, 336 total of positions were present. Evolutionary analyses were conducted in MEGA6 (Tamura et al., 2013).

### 2.6 Phylogenetic analysis

For phylogenetic analysis of goat IL‐33 gene, the nucleotide sequences of the concerned gene were compared with that of sheep, humans, rat, mouse, horse, cattle, buffalo, rabbit, camel, dog, and cat to detect its orthologs. Phylogenetic tree was constructed after nucleotide sequences retrieval from NCBI. Further, the sequence was analyzed by using MEGA6 software package and neighbor‐joining technique was employed for constructing phylogenetic tree, while similarity index of genetic sequence was analyzed through ClustalW software of eight species for IL‐33 gene.

### 2.7 Codon‐based dN‐dS and neutrality test

In this study, maximum likelihood computations of dS (synonymous substitutions number in each site), dN (nonsynonymous substitutions), and their ratios were estimated by using HyPhy software package (Pond & Muse, 2005). dN/dS ratio is a useful measure for determining the codons for positive selection. A total of 336 positions in the final dataset were determined. Both the analysis involved twelve nucleotide sequences. All positions containing gaps and missing data were eliminated; thereafter, evolutionary analyses were performed in MEGA6 (Tamura et al., 2013). Higher value of dN/dS ratio is an indication of positive values and vice versa. In other words, higher existence of dN shows more positive values. For neutrality test, the variance of difference was compared using the analytical method. Thereafter, analyses were conducted by using the Nei‐Gojobori method (Nei & Kumar, 2000).

## 3 Results

**Figure 1:**
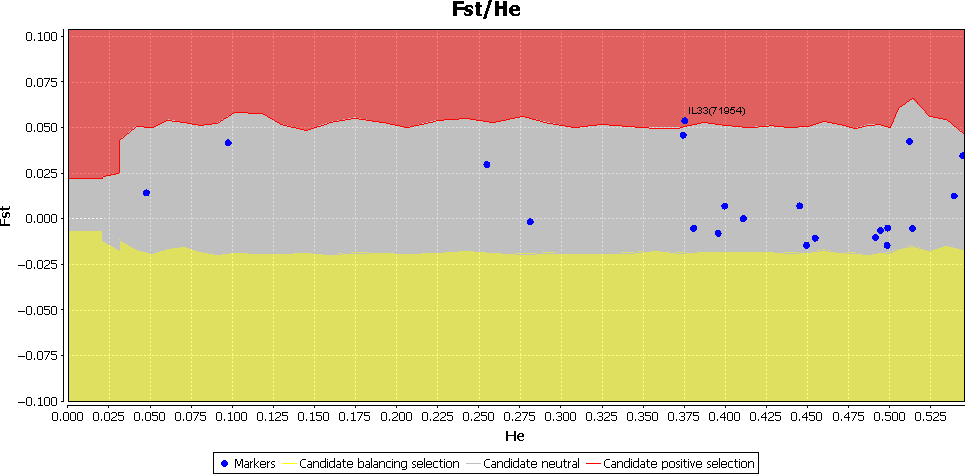
Candidate gene IL‐33 under positive selection keeping the 95% confidence interval. *F*_ST_ (Fixation index), *H*_e_ (Heterozygosity)

Of four goat breeds, the sequence description revealed 21 SNPs from 10 loci which were genotyped for advance analysis. The recognition of IL‐33 (71954) loci was done through *F*_ST_‐based technique similar to selection sweep in the other studied breeds. The outlier approach of FDIST method assisted in positive selection of IL‐33 (71954) gene. Through Lositan FDIST examination, the statistical location of this gene was outside the 95% confidence interval of *F*_ST_ and the conditional combined allocation of mean heterozygosity (Figure 1). Thus, via positive selection, the IL‐33 (71954) gene was found to be significantly (*p* < .05) in the region resultant (Table 1).

**Table 1:** Locus, heterozygosity (*H*_e_), and Fixation Index (*F*_ST_) for each of 21 genotyped SNPs

Locus	Het	*F*_ST_	*p*(Simul *F*_ST_ < sample *F*_ST_)
IL‐3	0.28	−0	0.44
DRB‐1	0.25	0.03	0.87
DRB‐1	0.4	0.01	0.6
GDF‐9	0.4	−0.01	0.26
IFNG	0.54	0.03	0.95
IGF‐1	0.05	0.01	0.69
IGF‐1	0.45	−0.01	0.1
IL1‐α	0.51	−0.01	0.31
IL1‐α	0.38	−0.01	0.34
IL1‐β	0.49	−0.01	0.21
IL1‐β	0.5	−0	0.4
IL1‐β	0.41	0	0.49
IL‐8	0.51	0.04	0.91
IL‐8	0.54	0.01	0.72
IL‐31	0.5	−0.01	0.11
IL‐33(63854)	0.49	−0.01	0.36
IL‐33(70589)	0.1	0.04	0.95
IL‐33(71954)	0.38	0.05	0.98*
IL‐32	0.37	0.05	0.96
IL‐32	0.45	−0.01	0.18
IL‐32	0.44	0.01	0.6

Heterozygosity (*H*_e_) and Fixation Index (*F*_ST_) *p*(Simulated *F*_ST_ < sample *F*_ST_).

*indicate significance level.

### 3.1 Positive selection of IL‐33 gene by FDIST analysis

The location of IL‐33 (71954) gene was in the outlier area while considering 95% confidence interval in Lositan FDIST analysis, with mean heterozygosity and provisional combined allocation of *F*_ST_ (Figure 1). Moreover, the presence of IL‐33 (71954) gene selected positively was significantly (*p *<* *.05) located in equivalent positive selection area (Table 1).

### 3.2 Evolutionary analysis of positive selection models

The extra replacements of nonsynonymous over synonymous depicted a molecular signal for positive selection. The BIC model has lowest scores (Bayesian information criterion) that are measured to explain the substitution outline the best. The lowest value of BIC is 4252.78, and the highest value is 4368.553. For all models, lowest value of AICc (Akaike information criterion, corrected) is 4101.827 and highest value is 4236.441, maximum likelihood value (*lnL*) is in the range of −2026.764 to −2097.105, and the number of considerations (including branch lengths) is also in the range of 21 to 31. Nonuniformity of evolutionary rates among sites may be modeled by using a discrete Gamma distribution (+G): The lowest value is 1.21 and the highest is 4.52 with five rate categories, and by assuming that a certain fraction of sites is evolutionarily invariable (+I): The lowest value is 0.15 and the highest is 0.28. Every time applicable, approximation of gamma profile parameter and/or the predictable fraction of invariant sites are revealed. Assumed values of transition/transversion bias (R) are in range of 0.50 to 1.62 for all models, as well. They are pursued by rates of base substitutions (r), nucleotide frequencies (f) for all pairs of nucleotide. Comparative values of immediate r should be measured when estimated them. For competence, sum of r values is made equal to 1 for each model (Table 2).

**Table 2:** Different models for positive selection tests for IL‐33 gene

Model	Parameters	BIC	AICc	*lnL*	(+I)	(+G)	R	*f*(A)	*f*(T)	*f*(C)	*f*(G)	r(AT)	r(AC)	r(AG)	r(TA)	r(TC)	r(TG)	r(CA)	r(CT)	r(CG)	r(GA)	r(GT)	r(GC)
T92 + G	24	4252.78	4101.83	−2026.76	n/a	1.27	1.6	0.29	0.29	0.21	0.21	0.05	0.04	0.13	0.05	0.13	0.04	0.05	0.18	0.04	0.18	0.05	0.04
T92 + I	24	4253.94	4102.993	−2027.35	0.27	n/a	1.55	0.29	0.29	0.21	0.21	0.06	0.04	0.13	0.06	0.13	0.04	0.06	0.18	0.04	0.18	0.06	0.04
K2 + G	23	4254.4	4109.725	−2031.72	n/a	1.21	1.6	0.25	0.25	0.25	0.25	0.05	0.05	0.15	0.05	0.15	0.05	0.05	0.15	0.05	0.15	0.05	0.05
K2 + I	23	4255.36	4110.685	−2032.2	0.28	n/a	1.56	0.25	0.25	0.25	0.25	0.05	0.05	0.15	0.05	0.15	0.05	0.05	0.15	0.05	0.15	0.05	0.05
T92 + G+I	25	4260.16	4102.933	−2026.3	0.18	3.13	1.58	0.29	0.29	0.21	0.21	0.06	0.04	0.13	0.06	0.13	0.04	0.06	0.18	0.04	0.18	0.06	0.04
K2 + G+I	24	4261.62	4110.67	−2031.19	0.19	3.21	1.59	0.25	0.25	0.25	0.25	0.05	0.05	0.15	0.05	0.15	0.05	0.05	0.15	0.05	0.15	0.05	0.05
HKY+G	26	4269.73	4106.229	−2026.94	n/a	1.25	1.61	0.308	0.272	0.214	0.206	0.05	0.04	0.13	0.06	0.13	0.04	0.06	0.17	0.04	0.19	0.05	0.04
TN93 + G	27	4270.59	4100.808	−2023.22	n/a	1.26	1.62	0.308	0.272	0.214	0.206	0.05	0.04	0.1	0.06	0.17	0.04	0.06	0.21	0.04	0.15	0.05	0.04
HKY+I	26	4271.03	4107.528	−2027.59	0.27	n/a	1.56	0.308	0.272	0.214	0.206	0.05	0.04	0.13	0.06	0.13	0.04	0.06	0.17	0.04	0.19	0.05	0.04
TN93 + I	27	4272.92	4103.146	−2024.38	0.26	n/a	1.56	0.308	0.272	0.214	0.206	0.05	0.04	0.1	0.06	0.16	0.04	0.06	0.2	0.04	0.15	0.05	0.04
HKY+G+I	27	4277.15	4107.374	−2026.5	0.18	3.04	1.59	0.308	0.272	0.214	0.206	0.05	0.04	0.13	0.06	0.13	0.04	0.06	0.17	0.04	0.19	0.05	0.04
TN93 + G+I	28	4278.23	4102.177	−2022.89	0.15	2.51	1.61	0.308	0.272	0.214	0.206	0.05	0.04	0.1	0.06	0.17	0.04	0.06	0.21	0.04	0.15	0.05	0.04
T92	23	4282.43	4137.759	−2045.74	n/a	n/a	1.48	0.29	0.29	0.21	0.21	0.06	0.04	0.13	0.06	0.13	0.04	0.06	0.18	0.04	0.18	0.06	0.04
K2	22	4286.94	4148.549	−2052.15	n/a	n/a	1.48	0.25	0.25	0.25	0.25	0.05	0.05	0.15	0.05	0.15	0.05	0.05	0.15	0.05	0.15	0.05	0.05
GTR+G	30	4289.51	4100.918	−2020.23	n/a	1.28	1.63	0.308	0.272	0.214	0.206	0.04	0.05	0.1	0.04	0.17	0.03	0.08	0.21	0.05	0.15	0.04	0.05
GTR+I	30	4292.74	4104.144	−2021.84	0.26	n/a	1.57	0.308	0.272	0.214	0.206	0.04	0.05	0.1	0.05	0.16	0.03	0.08	0.2	0.05	0.15	0.04	0.05
GTR+G+I	31	4297.43	4102.564	−2020.03	0.12	2.19	1.62	0.308	0.272	0.214	0.206	0.04	0.05	0.1	0.04	0.17	0.03	0.08	0.21	0.05	0.15	0.04	0.05
TN93	26	4300.35	4136.848	−2042.25	n/a	n/a	1.49	0.308	0.272	0.214	0.206	0.05	0.04	0.1	0.06	0.16	0.04	0.06	0.2	0.04	0.15	0.05	0.04
HKY	25	4300.37	4143.146	−2046.41	n/a	n/a	1.48	0.308	0.272	0.214	0.206	0.05	0.04	0.12	0.06	0.13	0.04	0.06	0.16	0.04	0.19	0.05	0.04
GTR	29	4318.23	4135.905	−2038.74	n/a	n/a	1.49	0.308	0.272	0.214	0.206	0.04	0.06	0.1	0.05	0.16	0.03	0.08	0.2	0.05	0.15	0.04	0.05
JC+I	22	4340.13	4201.74	−2078.74	0.27	n/a	0.5	0.25	0.25	0.25	0.25	0.08	0.08	0.08	0.08	0.08	0.08	0.08	0.08	0.08	0.08	0.08	0.08
JC+G	22	4340.4	4202.007	−2078.88	n/a	1.33	0.5	0.25	0.25	0.25	0.25	0.08	0.08	0.08	0.08	0.08	0.08	0.08	0.08	0.08	0.08	0.08	0.08
JC+G+I	23	4347.15	4202.476	−2078.1	0.21	4.52	0.5	0.25	0.25	0.25	0.25	0.08	0.08	0.08	0.08	0.08	0.08	0.08	0.08	0.08	0.08	0.08	0.08
JC	21	4368.55	4236.441	−2097.11	n/a	n/a	0.5	0.25	0.25	0.25	0.25	0.08	0.08	0.08	0.08	0.08	0.08	0.08	0.08	0.08	0.08	0.08	0.08

Models with the lowest BIC scores (Bayesian information criterion) are considered to describe the substitution pattern the best. For each model, AICc value (Akaike information criterion, corrected), maximum likelihood value (lnL), and the number of parameters (including branch lengths) are also presented. Nonuniformity of evolutionary rates among sites may be modeled by using a discrete Gamma distribution (+G) with 5 rate categories and by assuming that a certain fraction of sites are evolutionarily invariable (+I). Whenever applicable, estimates of gamma shape parameter and/or the estimated fraction of invariant sites are shown. Assumed or estimated values of transition/transversion bias (R) are shown for each model, as well. They are followed by nucleotide frequencies (f) and rates of base substitutions (r) for each nucleotide pair. Relative values of instantaneous r should be considered when evaluating them. For simplicity, sum of r values is made equal to 1 for each model. For estimating ML values, a tree topology was automatically computed. The analysis involved 12 nucleotide sequences. Codon positions included were 1st+2nd+3rd+Noncoding. All positions containing gaps and missing data were eliminated. There were a total of 336 positions in the final dataset. Evolutionary analyses were conducted in MEGA6.

### 3.3 Phylogenetic relationship of IL‐33 gene among species

**Figure 2:**
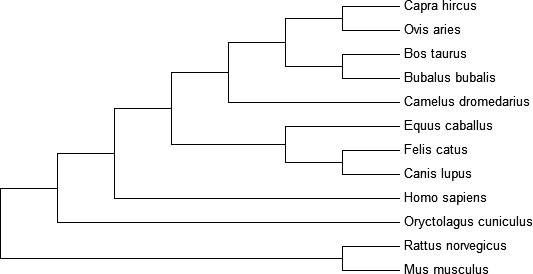
Phylogenetic relationship of IL‐33 gene among species

The thorough coding sequences of 12 different mammalian species for IL‐33 gene were gathered, keeping goat as reference species from the public database GenBank. The alignment of obtained sequences plus their respective amino acids was done through using MEGA6.0 software. Annual editing of all the gained sequences was developed before phylogenetic analysis through this software. The caprine IL‐33 gene was found closely related with that of sheep, camel, bison, cattle, buffalo, cat, horse, humans, rabbit, and dog (Figure 2). Nucleotide sequences having genetic similarity of goat IL‐33 gene were more closer to sheep (98.74%), buffalo (94.56%), cattle (93.74%), camel (86.55%), cat (80.81%), humans (80.1%), horse 79.97, dog (79.7%), rabbit (71.93%), mouse (66.57%), and rat (66.42%), respectively, shown in Table 3.

**Table 3:** Coding sequences similarity analysis and values of pair wise comparison of IL‐33 gene among different species

	Goat	Sheep	Cattle	Buffalo	Humans	Camel	Rabbit	Mouse	Rat	Cat	Dog	Horse
Goat		98.74	93.74	94.56	80.1	86.55	71.93	66.57	66.42	80.81	79.7	79.97
Sheep	98.74		92.72	93.58	79.76	86.13	71.52	66.72	66.12	80.23	79.11	79.26
Cattle	93.74	92.72		96.57	79.07	88.36	70.16	67.94	66.87	81.42	80.37	81.42
Buffalo	94.56	93.58	96.57		80.79	87.83	72.65	69.02	67.88	82.52	81.48	81.84
Humans	80.1	79.76	79.07	80.79		79.85	76.7	72.59	72.2	80.97	80.27	78.47
Camel	86.55	86.13	88.36	94.56	79.85		71.72	68.04	67.14	83.02	82.2	82.47
Rabbit	71.93	71.52	70.16	72.65	76.7	71.72		67.78	66.01	71.08	72.07	70.78
Mouse	66.57	66.72	67.94	69.02	72.59	68.04	67.78		88.43	68.75	67.42	66.84
Rat	66.42	66.12	66.87	67.88	72.2	67.14	66.01	88.43		68.77	67.03	67.89
Cat	80.81	80.23	81.42	82.52	80.97	83.02	71.08	68.75	68.77		88.54	82.33
Dog	79.7	79.11	80.37	81.48	80.27	82.2	72.07	67.042	67.03	88.54		80.64
Horse	79.97	79.26	81.42	81.84	78.47	82.47	70.78	66.84	67.89	82.33	80.64	

Similar values under triangle (%) in different species.

### 3.4 Determination of dN‐dS

**Figure 3:**
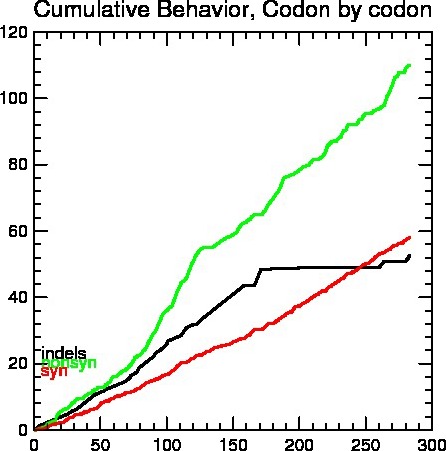
The graphical distributions of synonymous and nonsynonymous codon changes are counted

For each codon, estimates of the numbers of inferred synonymous (s) and nonsynonymous (n) substitutions are presented along with the numbers of sites that are estimated to be synonymous (S) and nonsynonymous (N). The test statistic dN‐dS is used for detecting codons that have undergone positive selection, where dS is the number of synonymous substitutions per site (s/S) and dN is the number of nonsynonymous substitutions per site (n/N). A positive value for the test statistic indicates an overabundance of nonsynonymous substitutions (Table 4). A total of nine triplet codons that were found at different positions have been elaborated as Syn (s), nonsyn (n), Synsites (S), Nonsynsites (N), dS, dN, and dN‐dS. The ranges of synonymous (s) and nonsynonymous (n) substitutions are (0) and (3–5), respectively, whereas the estimated site of synonymous (S) and nonsynonymous (N) was present in the range of (0.34–1) and (1.49–2.65), respectively. The values of dN‐dS are positive selected sites >1 which is resulted from the subtraction of dN nonsynonymous substitutions per site (n/N) to dS synonymous substitutions per site (s/S). The values of dN‐dS ranged from 1.13 to 2.21 (Table 4). The overall graphical distribution of synonymous and nonsynonymous codon changes is counted, as well as the number of potential synonymous and nonsynonymous changes when comparing sequences. Ambiguous codons or codons with insertions are excluded from the all of the compared codons. The overall sequence distances are calculated as well as codon‐by‐codon distances are summarized in Figure 3.

**Table 4:** Maximum likelihood analysis of natural selection codon by codon

Codon#	CodonStart	Triplet	Syn (s)	Nonsyn (n)	Synsites (S)	Nonsynsites (N)	dS	dN	dN‐dS
12	517	GAA	0	4	0.38	2.42	0	1.65	1.65
17	532	TTA	0	3	1	1.49	0	2.01	2.01
24	556	AGT	0	3	0.35	2.65	0	1.13	1.13
26	562	CGG	0	3	0.49	2.19	0	1.37	1.37
56	658	AAC	0	5	0.47	2.5	0	2	2
60	670	GAA	0	3	0.34	2.59	0	1.16	1.16
69	697	ACG	0	5	0.41	2.26	0	2.21	2.21
100	802	GAT	0	5	0.38	2.61	0	1.92	1.92
107	823	ACA	0	5	0.44	2.54	0	1.97	1.97

(S)Number of synonymous sites in a sequence, (N) Number of nonsynonymous sites in a sequence, (dS) Number of synonymous substitutions per synonymous site, (dN) Number of nonsynonymous substitutions per nonsynonymous site. ω = (dN‐dS) values >1 indicating significance.

### 3.5 Determination of neutrality for analysis between sequences

The probability of rejecting the null hypothesis of strict neutrality (dN = dS) (below diagonal) is shown. Values of *p* < .05 are considered significant at the 5% level and are highlighted (Table 5). The test statistic (dN‐dS) is shown above the diagonal. dS and dN are the numbers of synonymous and nonsynonymous substitutions per site, respectively. In the neutrality analysis of different species, the mouse has more significant values 0.001 in humans, cat has 0.002, 0.001, 0.002, and 0.001, dog has 0.003, 0.002, 0.005, 0.002, and 0.012, camel has 0.001 and 0.007, and horse has 0.024, 0.015, 0.049, 0.004, 0.013, 0.006, and 0.039, but other species did not show any significance.

**Table 5:** Codon‐based test of neutrality for analysis between sequences

	Goat	Sheep	Cattle	Humans	Rat	Mouse	Buffalo	Rabbit	Cat	Dog	Camel	Horse
Goat		−0.420	−0.587	−3.995	−6.580	−5.449	−1.347	−4.307	−3.234	−2.981	−0.939	−2.282
Sheep	0.675		−0.716	−4.164	−6.916	−5.758	−1.403	−4.473	−3.406	−3.158	−1.205	−2.462
Cattle	0.558	0.475		−3.568	−6.316	−5.242	−0.216	−3.997	−3.155	−2.835	−0.727	−1.985
Humans	0.000	0.000	0.001^a^		−4.384	−4.925	−3.647	−5.861	−3.803	−3.897	−4.063	−2.956
Rat	0.000	0.000	0.000	0.000		−6.119	−5.786	−8.413	−5.723	−6.134	−6.723	−5.276
Mouse	0.000	0.000	0.000	0.000	0.000		−4.730	−6.807	−5.910	−6.760	−4.929	−5.721
Buffalo	0.181	0.163	0.829	0.000	0.000	0.000		−4.129	−3.531	−3.219	−1.293	−2.520
Rabbit	0.000	0.000	0.000	0.000	0.000	0.000	0.000		−5.393	−4.055	−3.709	−3.670
Cat	0.002^a^	0.001^a^	0.002^a^	0.000	0.000	0.000	0.001^a^	0.000		−2.552	−3.526	−3.852
Dog	0.003^a^	0.002^a^	0.005^a^	0.000	0.000	0.000	0.002^a^	0.000	0.012^a^		−2.740	−2.778
Camel	0.349	0.231	0.468	0.000	0.000	0.000	0.198	0.000	0.001^a^	0.007^a^		−2.086
Horse	0.024^a^	0.015^a^	0.049^a^	0.004^a^	0.000	0.000	0.013^a^	0.000	0.000	0.006^a^	0.039^a^	

a Significance of nutrality, respectively.

### 3.6 Analysis of antigenic domains of IL‐33

**Figure 4:**
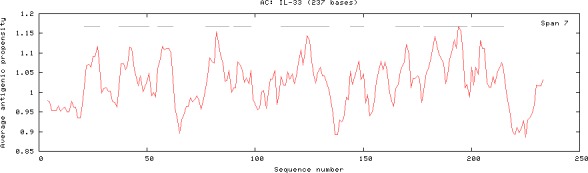
Antigenic domains of IL‐33 in goat

**Figure 5:**
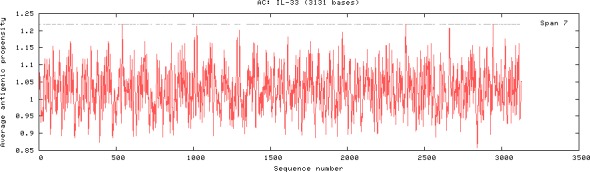
Antigenic domains of IL‐33 in 12 species

The antigenic domains of goat and all 12 animal species were predicted by (Kolaskar & Tongaonkar, 1990) method using online software tool (http://imed.med.ucm.es/tool/antigenic.pl). The results revealed that 11 antigenic domains of IL‐33 from goat lie from 4th to 225th amino acids (Figure 4) and 143 antigenic domains for 12 animal species including goat lie from 4th to 3114th amino acids (Figure 5). The average antigenic propensity for goat IL‐33 of 247 amino acid residues was 1.0256 and that for all 12 animal species of 3,131 amino acid residues was 1.0273. These predictions may assist the selection of goat IL‐33 antigenic epitopes to enable the preparation of antibodies for use in testing tissue distribution of IL‐33.

## 4 Discussion

The current improvement in the significant directories of genetic difference has projected novel implication for the innovation of positive selection targets, which finally would be helpful to clarify the drift and selection roles in evolutionary methods. Moreover, signatures of positive selection hinder the genome region that functionally plays significant role. Therefore, the determination of such genomic regions will present significant associate for the identification of genetic variation, which ultimately would initiate the intervention of these efficient genomic regions and expansion of phenotypic collection. Hence, the population history is designed through chronological actions and services which provide information for the target of positive selection.

The genetic ground of various traits of different species has been formulated by candidate gene approach. Candidate gene discovery has a vital role in livestock populations' phenotypic difference that provides new information about evolutionary process and positive selection (Brown et al., 2013). The present study of IL‐33 (71954) gene was foretold by *F*_ST_ and operating the mean heterozygosity. Hence, this study proved the important gene and forecast the loci connected with various complications of diseases in Chinese goat population.

Earlier, many genes correlated with positive selection were identified by *Lnl*. The genes selected by *Lnl* are revealed as true cases for adaptation that might engage in positive selection (Anisimova, Nielsen, & Yang, 2003). In the present study, likelihood logs were used for the determination of *Lnl,* and different models were implemented. According to positive selection, ω = dN − dS > 1 value which are concerned in positive selection. Therefore, IL‐33 gene evolutionary study in 12 species of mammals is evident in purified selection and previously no reports are designed for positive selection like that.

The outlier loci documented revealed adaptive variability in the studied species attributed by extensive climate difference in their local areas and energetic import/export which recommend their capability of adjusting to a new environment. The FSHβ gene is extremely under strong positive selection for the development and proper functionality of gonads (Ijaz et al., 2015). The current research study is in strong favor of the results in positive selection.

After assessment of nonsynonymous and synonymous mutations of DRB‐1 gene in Eastern woodchuck represents a powerful evidence for positive selection, which shows that the DRB‐1 gene is under positive selection (Klein, 1986). Here, this study significantly supported that IL‐33 gene of our study is also under positive selection in goat. Several nonsynonymous mutations were found around or within the putative antigen‐binding sites. Moreover, these analyses show that the positive selection sites were functionally important (Bernatchez & Landry, 2003). In the current study, the IL‐33 found to be nonsynonymous and also under positive selection. The change in amino acid (threonine to asparagine) in IL‐33 gene may be helpful for the positive selection and may also change the function of the gene. It might play a crucial role in immune system to control the diseases or disorders.

Bustamante discovered that minor amount of nonsynonymous substitution in genes was found significantly associated with Mendelian disease (Bustamante et al., 2005); however, many genes recognized as a potential position for positive selection were assessed as a cause of cancer process (Nielsen et al., 2005). Genes that are associated with complex diseases, like asthma interleukin‐13 (Fullerton et al., 2002), IL‐4 (Rockman, Hahn, Soranzo, Goldstein, & Wray, 2003; Sakagami et al., 2004) and IL‐1A (Akey et al., 2004), [cytochrome P450 (CYP3A) (Thompson et al., 2004), cardiovascular disease [matrix metallopeptidase 3(MMP3) (Rockman et al., 2003), and (AGT) (Nakajima et al., 2004) type 2 diabetes (CAPN10 (Fullerton et al., 2002)] have also reported as positive signature selection in different studies. So selection of genes may play a key role in disease mapping via genomewide analysis. The current study coincides with several previous studies of positive signature selection about different genes. IL‐33 also selected as positive signature might be helpful in genetic control of different diseases with the enhancement of immune system.

Asif et al. (2016) worked on gene IL‐32‐positive selection and different phylogenetic analyses in different animals. Interleukin (IL)‐32 is documented as pro‐inflammatory cytokine that plays substantial role in various biological processes, whereas the role of IL‐33 has been predictable in various diseases, such as cardiovascular diseases (Miller & Liew, 2011), especially allergic asthma (Liew, 2012; Saluja et al., 2015), allergies, chronic inflammation of the gut (Lopetuso et al., 2013), disorders of the central nervous system (Gadani et al., 2015), and rheumatoid arthritis (Palmer & Gabay, 2011). The function of IL‐33 is different from IL‐32; however, the positive selection of IL‐32 supports the current study of IL‐33.

However, the phylogeny of goat interleukin‐33 is poorly described in scientific reports. Therefore, complete coding sequences were compared in different mammalian species and found that the IL‐33 gene of goat shared 98.74% identity with sheep, 66.42% with rat, 66.57% mouse, 93.74% cattle, 94.56% buffalo, 79.97% horse, 86.55% camel, 80.1% humans and 79.7% dog. Hence, in this study, after construction of a phylogenetic tree, it is found that goat IL‐33 has a close resemblance to sheep IL‐33, and therefore, they were placed in the same phylogenetic group.

## 5 Conclusion

The alpha and omega of the whole story concludes here that the methodologies designed to explore the nucleotide and genetic variations in this research lead to the better thoughtfulness of the positive selection of IL‐33 gene single‐nucleotide polymorphism and molecular evolution. Hence, our study provided novel information about the nucleotide variations in IL‐33 gene and also found to be nonsynonymous which may change function of gene which plays a crucial role in the genetic control of diseases in local Chinese goat breeds.

## Conflict of Interest

None declared.
